# Granulocytic Myeloid Derived Suppressor Cells Expansion during Active Pulmonary Tuberculosis Is Associated with High Nitric Oxide Plasma Level

**DOI:** 10.1371/journal.pone.0123772

**Published:** 2015-04-16

**Authors:** Sary El Daker, Alessandra Sacchi, Massimo Tempestilli, Claudia Carducci, Delia Goletti, Valentina Vanini, Vittorio Colizzi, Francesco Nicola Lauria, Federico Martini, Angelo Martino

**Affiliations:** 1 Laboratory of Cellular Immunology, “Lazzaro Spallanzani” National Institute for Infectious Diseases, Rome, Italy; 2 Unité de Biologie des Populations Lymphocytaires, Department of Immunology, Institut Pasteur, Paris, France; 3 Department of Biology, University of Rome Tor Vergata, Rome, Italy; 4 Clinical Biochemistry and Pharmacology Laboratory, “Lazzaro Spallanzani” National Institute for Infectious Diseases, Rome, Italy; 5 Department of Experimental Medicine, Sapienza University of Rome, Rome, Italy; 6 Translational Research Unit, “Lazzaro Spallanzani” National Institute for Infectious Diseases, Rome, Italy; 7 Respiratory Infectious Diseases Unit, "Lazzaro Spallanzani" National Institute for Infectious Diseases, Rome, Italy; National Institute of Infectious Diseases, JAPAN

## Abstract

Tuberculosis (TB) is still the principal cause of death caused by a single infectious agent, and the balance between the bacillus and host defense mechanisms reflects the different manifestations of the pathology. The aim of this work was to study the role of myeloid-derived suppressor cells (MDSCs) during active pulmonary tuberculosis at the site of infection. We observed an expansion of MDSCs in the lung and blood of patients with active TB, which are correlated with an enhanced amount of nitric oxide in the plasma. We also found that these cells have the remarkable ability to suppress T-cell response, suggesting an important role in the modulation of the immune response against TB. Interestingly, a trend in the diminution of MDSCs was found after an efficacious anti-TB therapy, suggesting that these cells may be used as a potential biomarker for monitoring anti-TB therapy efficacy.

## Introduction

Tuberculosis (TB) is one of the biggest killers among infectious diseases, despite the worldwide use of a live attenuated vaccine (BCG) and chemotherapy. This is principally due to the ability of the pathogen to evade the immune defenses of the host [1]. After entry into the body, the tubercle bacillus can interact with different cells of both innate and adaptive immune compartments. All together, these cells play an important role in the modulation and development of the pathology [2]. The different evasion mechanisms used by the pathogen include the arrest of phagolysosome maturation [3], resistance against reactive nitrogen intermediate (RNI), Nitric Oxide (NO) [4], interference with MHC class II antigen presentation [5], and the block of autophagy [6–9]. *Mycobacterium tuberculosis* (MTB) can also inhibit the immune response indirectly, promoting, for example, the accumulation of regulatory T-lymphocytes (T-reg) at the site of infection [10]. These cells are able to reduce the frequency of MTB-responsive CD4+ and CD8+ T-cells producing INF- γ, thus allowing for the replication of mycobacteria. The immune suppressive activity is not only specific for regulatory T-lymphocytes. In the 1980s, a new cell population, distinct from T and NK cells, was described in tumor-bearing mice [11]. These cells are now known as myeloid-derived suppressor cells (MDSCs) and constitute a heterogeneous population of cells that proliferate during cancer and other pathological processes such as traumatic stress, sepsis, acute inflammation, and bacterial, viral and parasitic infections [12]. These cells, defined in humans as CD11b+CD14-CD33+CD15+ and HLA-DR^low^ (granulocytic MDSCs), or CD11b+CD14+CD33+ and HLA-DR^low^ (monocytic MDSCs), are known to have the remarkable ability to suppress T-cell responses through diverse mechanisms, one of which is associated with the NO production through the L-arginine metabolism [13, 14, 15]. Little is known about the role of these cells during MTB infection [16, 17]. Recently, the expansion of MDSCs was observed in the blood of patients with pulmonary TB, and in the pleural fluid of extra-pulmonary pleural TB [18]. In this study, we evaluated the presence of MDSCs in the bronco-alveolar lavage (BAL) and blood of pulmonary TB patients, compared with healthy donors. In active TB, we found an expansion of MDSCs in both compartments, correlated with enhanced L-arginine catabolism and NO production. Considering the inability of immune cells to block MTB replication, especially at the first site of interaction [19], this could be an important piece of the puzzle to better understand the immunological environment during MTB infection and disease.

## Materials and Methods

### 2.1 Patient Population

Thirty subjects with active TB and fifteen healthy donors (HD) were enrolled at the respiratory disease wards at the “Lazzaro Spallanzani” National Institute for Infectious Diseases in Rome. All enrolled subjects were HIV-uninfected and not undergoing immune-suppressive therapy. The characteristics are reported in Table 1. All study participants had newly diagnosed pulmonary TB with a sputum culture-positive for Mtb on mycobacteria growth indicator tube culture, with confirmation through polymerase chain reaction. Patients with active TB were studied within 7 days of admission, before and after starting anti-TB therapy. Household contact TB subjects were exposed to *M*. *tuberculosis* infection by sharing air space with an index case who was sputum smear-positive and tuberculin skin test-positive (TST+). BAL was performed by instilling a sterile isotonic saline solution (4 times 30 ml) into an affected lung segment. Ten patients were also recruited after undergoing anti-TB treatment. Active TB patients were successfully treated with isoniazid, rifampicin, pyrazinamide and ethambutol for 2 months, then with isoniazid and rifampicin alone for a further 4 months. At the end of therapy, all TB patients were sputum culture-negative and defined as “cured TB”. We defined “end of therapy” patients as those evaluated 6 months after successful therapy. We defined “past TB” patients as those with culture-confirmed TB evaluated at least one year after having completed successful therapy. In addition, seven subjects with pulmonary infections not correlated with TB (sputum culture-negative for Mtb) were enrolled as non TB controls for BAL analysis (no TB).

**Table 1:** Demographic and clinical characteristics of the individuals enrolled in the study.

				CURED TB		
	Healthy donors	TB Contacts	Active TB	End of therapy	After therapy	Total
					(> 1 year)	
N (%)	15 (22.4)	12 (18.0)	30(44.8)	5(7.5)	5(7.5)	67(100)
Male gender (%)	7(46.7)	8(57.1)	19(63.3)	1(10)	4(40)	39(58.2)
BCG-vaccinated	na	na	30	4	3	37
anti-TB treatment	-	-	-	5	5	10
Smokers (%)	na	na	16(53.3)	0 (0)	3 (60)	-

na: not available

### 2.2 Cell preparation, flow cytometry and MDSC morphology

Peripheral blood mononuclear cells (PBMCs) from TB patients and healthy controls were isolated from whole blood using Ficoll density gradient centrifugation, as previously described [19]. Bronchoalveolar cells (BALc) from TB patients were processed in a BSL3 laboratory. Bronchoalveolar lavages were treated with Sputasol (Oxid) for 15 minutes at room temperature. After filtering with BD Falcon cell strainer 100 μm, BALc were washed twice in PBS 1X and suspended in FACSFlow (BD-Pharmagen). The following anti-human Abs were used: anti-CD11b (clone ICRF44), anti-CD14 (clone HCD14), anti-CD15 (clone W6D3), anti-CD33 (clone HIM3-4), anti-HLA-DR (clone L243), anti-CD3 (clone UCHT1). PBMCs and BALc were incubated for 15 min at 4°C with fluorochrome-conjugated mAb. Samples were washed in PBS 1X, fixed in paraformaldehyde (PFA 1% for PBMCs and 4% for BALc), suspended in FACSFlow and immediately acquired using FACS CANTO II flow cytometer (BD Biosciences). A total of 500,000 events were acquired for each sample and analyzed with CellQuest software (BD Biosciences). The HLA DR- CD11b+ CD33+ cells were sorted (FACS ARIA II, BD Biosciences) and cytospin slides of enriched cell fractions were prepared and stained with hematoxylin/eosin dye according to standard protocols.

### 2.3 Mixed lymphocyte reaction (MLR)

After isolating the PBMCs from the whole blood of TB patients and HD, the cells derived from HD were labeled with CFSE (according to the manufacturer’s protocol, Invitrogen) while PBMCs from TB patients were stained with CD14 FITC and CD11b PE. The CD11b+ CD14- cells were then depleted by sorting (FACS ARIA II, BD Biosciences) and MDSC-depleted PBMCs were treated with 0.5 mg/ml of mitomycin C (SIGMA) for 30 min at 37°C, 5% CO2. After extensive washings, 2x10^5^ CFSE-labeled PBMCs from healthy donors were co-cultured with 2x10^5^ MDSCs-depleted PBMCs from TB patients and different ratios of sorted MDSCs (1:2, 1:4 and 1:8). After 5 days, cells were harvested and stained with anti-CD3 mAb. The change in the CFSE signal of CD3+ T-cells was measured by flow cytometry as fluorescence intensity.

### 2.4 High-Performance Liquid Chromatography (HPLC)

Cells were removed from plasma by centrifugation for 15 minutes at 2,000 g. After the addition of the internal standard norvaline and sarcosine. the liquid component (plasma) was deproteinizated by ultra filtration using Amicon ultra 10K 0.5mL filters. The samples were then subjected to automated pre-column derivatization with o-phthalaldehyde (OPA) and 9-fluorenylmethylchloroformate (FMOC) using 1200 Autosampler (Agilent Technologies). The resulting mixture of primary amino acids as isoindole (OPA) derivates and secondary amino acids as FMOC derivates was injected onto the reversed-phase column and detected at two different wavelengths [20]. The separation and the fluorescence detection were done using a 1200 Series HPLC instrument (Agilent Technologies).

### 2.5 Nitrate/Nitrite colorimetric assay

The nitrate concentration in plasma samples was evaluated using the “Nitrate/Nitrite colorimetric assay kit” provided by Cayman, following the indicated protocol (cat.num. 780001). The absorbance was read at 540 nm using a plate reader (Victor, PerkinElmer).

### 2.6 Statistical analysis

Graph-Pad Prism version 4.00 for Windows was used to perform the statistical analysis. The Mann-Whitney test was used to assess differences between the different study groups. P values as <0.05 were considered significant.

### 2.7 Ethics Statement

The study was approved by the Ethics Committee of the “Lazzaro Spallanzani” National Institute for Infectious Diseases in Rome (Ethics Committee Number: 7/2010). Written informed consent was obtained from all the participants and clinical and functional data were collected on a standardized and anonymous collection form.

## Results

### 3.1 TB patients express high levels of MDSCs in both lung and blood compartments

**Fig 1:**
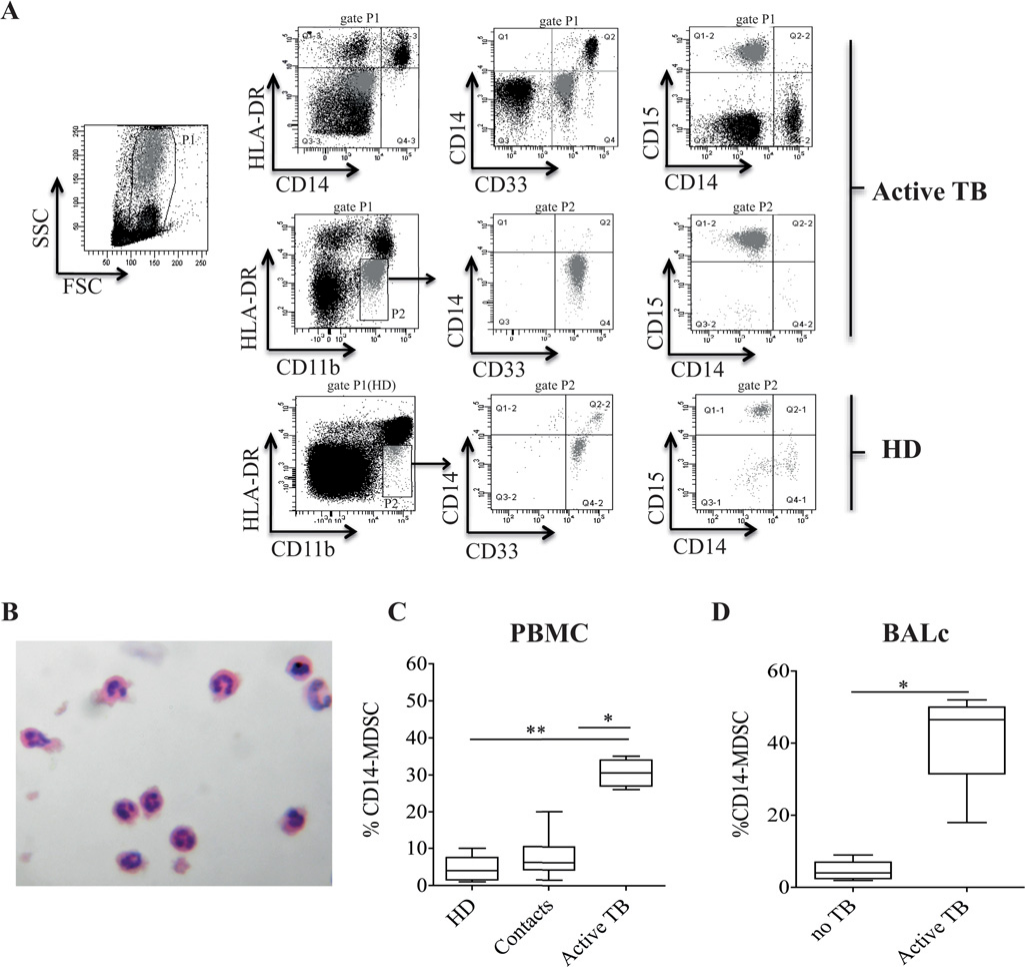
Frequency of granulocytic MDSCs in BALc and peripheral blood of active TB patients and HD. (A) Gating strategy used to analyze the frequency of MDSCs, and dot plots showing CD14 expression on cells from P1, bringing to front cells in P2 (MDSCs, gray). (B) MDSCs obtained by sorting were examined by light microscopy. Morphologic analyses of peripheral MDSCs showed substantial homogeneity, comprised only of subset exhibiting granulocytic characteristics. (C) MDSC frequency in PBMCs derived from healthy donors (N.15), contact TB (N.12) and active TB (N.30). (D) Frequency of MDSCs in BALc derived from TB patients (N.30) and individuals with pulmonary diseases not correlated with TB (No TB, N.8). Results are expressed as the median ± IQR. *P<0.05, **P<0.02.

Our aim was to evaluate whether the expansion of MDSCs previously observed in the peripheral blood from the pulmonary TB patients [18] also occurred in the lung. Therefore, the presence of MDSCs was evaluated in the PBMCs and BALc of TB patients by flow cytometry. MDSCs were identified as HLA-DR^-/low^CD11b+CD33+. Granulocytic and monocytic subsets were identified by using CD14 and CD15, and confirmed by Hematoxylin and Eosin staining (Fig 1A and 1B). A higher frequency of CD11b+CD14-CD33+CD15+HLA-DR^low/-^ MDSCs (CD14- MDSCs) was observed in PBMCs from TB patients compared to healthy donors (Fig 1A and 1C). Moreover, when household contacts were evaluated, the proportion of MDSCs was comparable to healthy controls (Fig 1C). A significant accumulation of the same MDSC subset was found in the BALc of pulmonary TB patients compared to pulmonary diseases uncorrelated with TB (Fig 1D). In our studies, we did not observe any difference between smokers and non-smokers regarding the frequency of MDSCs (data not shown).

### 3.2 MDSCs from TB patients suppress T-cell proliferation

**Fig 2:**
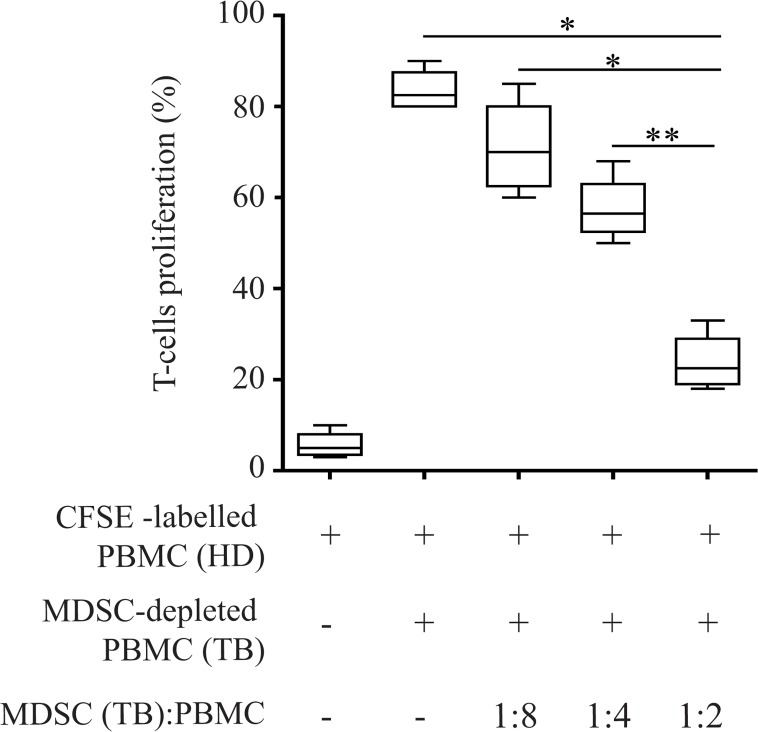
Peripheral blood CD14-CD11b+ MDSCs sorted by active TB patients suppress T-cell proliferation. PBMCs from TB patients were stimulated with CFSE labeled PBMC derived from healthy donors in the presence of sorted CD14^-^CD11b^+^ MDSCs at different ratios for 5 days. The percentage indicates the number of CD3+ T-cells which have undergone cellular division after 5 days. Results are expressed as the median ± IQR of three independent experiments. *P<0.05, **P<0.02.

We used a MLR experiment to test the ability of MDSCs derived from the blood of TB patients to suppress proliferation of T-cells derived from HD. MDSC-depleted PBMCs (TB), treated with mitomycin C, were cultured with allogeneic CFSE-labeled PBMCs from HD (CFSE-labeled PBMCs (HD)) and with sorted CD14-CD11b+CD33+ HLA-DR^low/-^ MDSCs (MDSCs (TB)) at different ratios (1:2, 1:4 and 1:8). We observed that while HD T cells proliferate in presence of allogeneic TB PBMC MDSC-depleted, the addition of MDSC population from TB patients dramatically suppressed the T-cells proliferation in a dose-dependent manner (Fig 2).

### 3.3 MDSC expansion is associated with low levels of Arginine and high levels of NO in the plasma of TB patients

**Fig 3:**
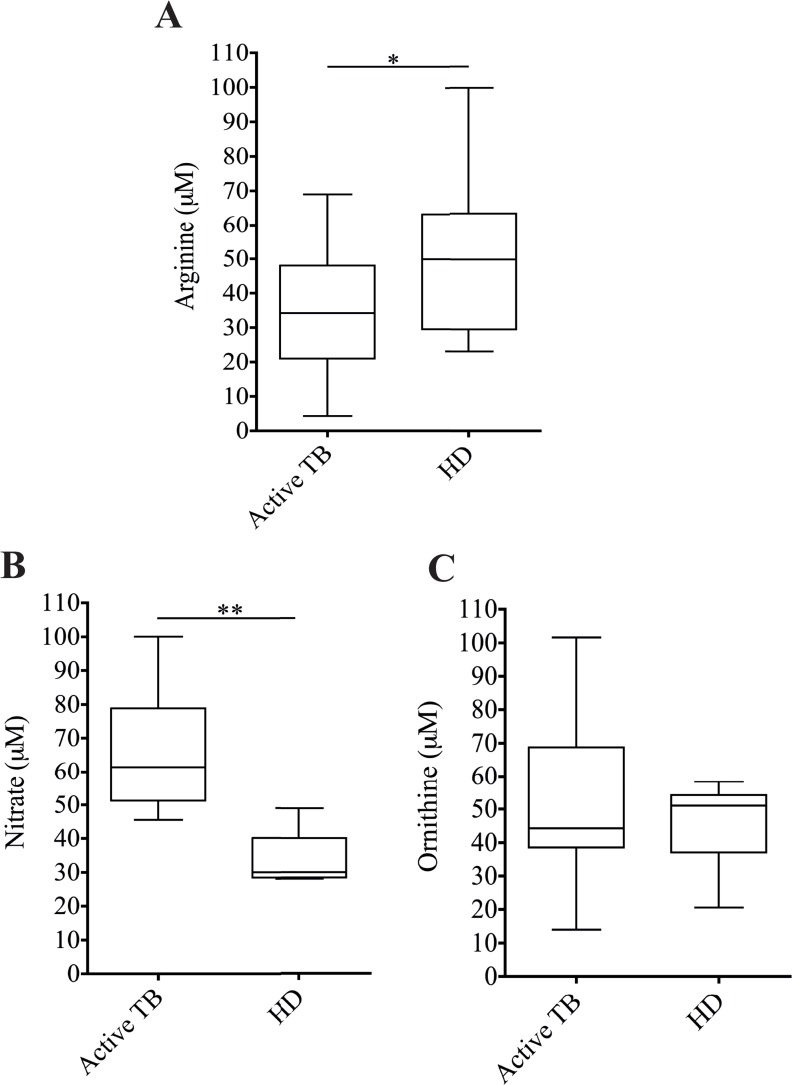
L-arginine metabolism is correlated with the inhibition activity of MDSCs. Levels of (A) L-arginine, (B) Ornithine and (C) Nitrate in the serum of healthy donors (HD, N.15) and active TB patients (TB, N.30). Results are expressed as the median ± IQR. *P<0.05 **P<0.02.

It was previously shown that MDSCs from TB patients may exert their suppressive function by arginase-I (Arg-I) [13–15]; however, a clear demonstration is missing. To evaluate whether Arg-I activity plays a role during TB infection, we tested the plasma level of its substrate L-arginine by HPLC. We observed that the concentration of this amino acid is lower in the plasma of TB patients compared to HD (Fig 3A), suggesting a higher activity of Arg-I. However, L-arginine serves as a substrate for two enzymes: *i*NOS, which generate NO, and arginase-I, which converts L-arginine to urea and L-ornithine. To evaluate which enzyme is involved in the arginine catabolism, we analyzed the concentration of NO and ornithine in the plasma of TB patients and healthy donors. We found a higher amount of NO in TB patients compared to HD (Fig 3B). On the contrary, no difference in the ornithine level between TB patients and healthy donors was observed (Fig 3C). These data indicate that in TB patients, *i*NOS is responsible for L-arginine degradation, suggesting that MDSCs could exert their suppression by *i*NOS activity.

### 3.4 The frequency of granulocytic MDSC is reduced after anti-TB treatment

**Fig 4:**
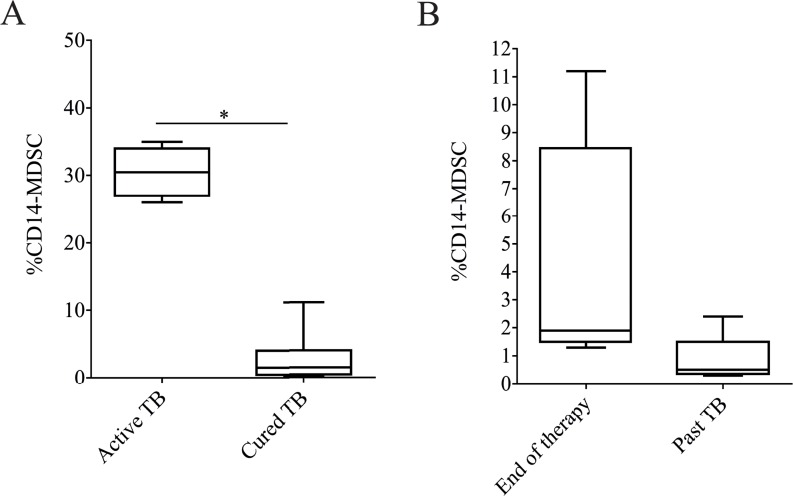
Successful TB treatment reduces the concentration of MDSCs in peripheral blood cells. (A) Analysis of MDSC frequency in patients with active TB (N.30) and cured TB (N.10). (B) MDSC frequency in cured TB at the end of therapy (N.5) and 1–3 years after the end of therapy (past TB, N.5). Results are expressed as the median ± IQR. *P<0.05.

We next assessed the effect of successful anti-TB therapy on the frequency of MDSCs. Therefore, we analyzed the proportion of CD14-MDSCs in PBMCs from patients with active TB, and patients after successful therapy. We found that in cured TB patients, the frequency of CD14-MDSCs was lower compared to active TB (Fig 4A). Furthermore, an almost significant decrease of MDSC frequency was found in patients 1–3 years after therapy compared to those at the end of therapy (Fig 4B) (p = 0.055). These data suggest that successful therapy associates with a decrease of MDSC frequency.

## Discussion

Natural suppressor cells in patients with cancer [11] were described for the first time more than 20 years ago, and their inhibition activity has been intensively studied in different pathological conditions. Recently, Obregon-Henao and colleagues [17] highlighted the suppressive role of Gr1^int^CD11b^+^ cells in mice models of *M*. *tuberculosis* infection. Moreover, Walzl’s group observed an induction of these cells during active pulmonary and pleural TB in humans [18]; in particular they found an expansion of MDSCs characterized by the capacity to reduce T-cell activation, alter T-cell trafficking, and suppress CD8(+) T-cell functions. In our study, we analyzed the proportion of MDSCs at the site of infection (BALc) and in the peripheral blood (PBMCs) of patients with active pulmonary TB. We confirmed an increased frequency of MDSCs in the peripheral blood of TB patients and, interestingly, we observed the same expansion in the BALc, where the immune system should perform its anti-MTB functions. In particular, the frequency of granulocytic subset was expanded in both compartments. Walzl’s group showed a high frequency of CD14+ MDSCs in pleural TB, suggesting that pulmonary and extra-pulmonary TB could induce different MDSC subset expansion. CD14 analysis was not performed in the pulmonary TB patients, even if a heterogeneous morphology (granulocytic and monocytic) was described by light microscopy on HLA-DR-/CD33+ isolated cells [18]. Conversely, in the present study, we did not find an expansion of MDSC subset frequency in PBMCs from household contacts. Since healthy contacts are sputum culture negative by definition, it is difficult to establish if they were harboring MTB or if the immune system efficiently cleared the infection. Thus, the role of MDSCs may vary in these different scenarios.

We also analyzed the frequency of MDSCs during MTB infection in patients before and after anti-TB treatment. We observed that successful therapy decreased the MDSC frequency in cured patients compared to those at diagnosis, confirming the central role played by MDSCs during TB disease. Interestingly, the lowest frequency of MDSCs was found in past TB patients, suggesting the potential use of these cells to monitor anti-TB treatment efficacy; however, larger studies are needed to validate this hypothesis.

In accordance with previous publications [18], we observed that MDSCs derived from TB patients are able to inhibit T-cell proliferation in a dose-dependent manner. Considering the heterogeneity of the mechanisms utilized by these cells to suppress T-cell activities in neoplastic and infectious diseases, we tried to identify the suppression mechanism used by these cells during TB. It has been previously shown that there is a trend of up-regulation of Arg-I activity in cultures containing MDSCs from individuals infected with MTB [18]. We observed a significant reduction of the concentration of L-arginine in plasma samples derived from TB patients. However, this reduction was associated with an equal concentration of ornithine, generated by the activity of Arg-I, and an up-regulation of NO production, generated by *i*NOS activity. All together these results suggest that one of the mechanisms used by the MDSCs of TB patients to suppress T-cell activity is nitric oxide- dependent.

It has been previously shown that the values of MTB-specific T-lymphocytes are 10-fold higher at the site of infection compared to the periphery [21]. On the other hand, a high frequency of MDSCs was found in pleural cells [18], and in the present study in BALc, suggesting that they may contribute to immune regulation in response to the inflammatory processes raised by MTB infection, rather than immune evasion.

In conclusion, our results confirm that MDSCs play a crucial role during MTB disease. The importance of these cells in immunomodulation, also recognized as one of the major mechanisms of tumor escape, is emerging with time. For this reason, several different therapeutic strategies that target these cells were adopted to avoid their suppressor activity. However, the exact role of MDSCs during TB is still not clear. In particular, further investigation is necessary to determine whether these cells are involved in MTB immune evasion or important in the containment of the TB immune pathogenesis. Understanding the role these cells play during TB infection could be key information for developing new approaches for therapeutic monitoring and therapy development.
